# Biventricular arrhythmogenic cardiomyopathy due to a *PKP2* mutation with severe heart failure and multifocal thromboembolism as the clinically apparent presentation: a case report

**DOI:** 10.1093/ehjcr/ytag636

**Published:** 2026-08-27

**Authors:** Binh Thi Thanh Dao, Thong Minh Chau

**Affiliations:** Faculty of Medicine, HUTECH University, 475A Dien Bien Phu Street, Thanh My Tay Ward, Ho Chi Minh City 72331, Vietnam; Department of Cardiology, 115 People's Hospital, 527 Su Van Hanh Street, Hoa Hung Ward, Ho Chi Minh City 72523, Vietnam

**Keywords:** Arrhythmogenic cardiomyopathy, *PKP2* mutation, Heart failure, Thromboembolism, Case report

## Abstract

**Background:**

Arrhythmogenic cardiomyopathy (ACM) is a genetic myocardial disease classically characterized by ventricular arrhythmias and right ventricular involvement. Biventricular variants involving *PKP2* mutations are increasingly recognized but rarely present primarily as severe heart failure and multi-focal thromboembolism.

**Case summary:**

A 22-year-old woman presented with progressive exertional dyspnoea and severe biventricular dysfunction [left ventricular systolic dysfunction (LVEF) 20%]. The clinical course was complicated by right subclavian vein thrombosis and an acute cerebellar infarction. Cardiac magnetic resonance imaging demonstrated biventricular involvement with a non-ischaemic pattern of myocardial fibrosis. Genetic testing identified a pathogenic heterozygous *PKP2* variant, which, integrated with the phenotype, established the diagnosis of ACM. Serial electrocardiographic assessment revealed a high burden of multifocal premature ventricular complexes, which persisted despite catheter ablation and increased during ambulation, indicating ongoing electrical instability. The patient received optimized guideline-directed medical therapy and rivaroxaban. At follow-up, she demonstrated reverse remodelling with an improved LVEF of 31%.

**Discussion:**

This case expands the clinical spectrum of *PKP2*-associated ACM, emphasizing that severe heart failure and thromboembolism can dominate the clinically apparent presentation. While significant arrhythmias, including non-sustained ventricular tachycardia and a high premature ventricular contraction (PVC) burden, are classic hallmarks of the disease, in this case, they did not prompt initial medical attention and were instead documented on later surveillance. Although electrical remodelling occurs early, it may remain clinically concealed until unmasked by progressive structural damage. Early integration of multimodality imaging and genetic testing is essential for accurate diagnosis and risk stratification. Implantable cardioverter-defibrillator implantation was planned for primary prevention of sudden cardiac death.

Learning points

- Arrhythmogenic cardiomyopathy may initially present with heart failure and thromboembolism before arrhythmias.
- Multimodality imaging and genetic testing are key to diagnosis.
- Arrhythmic burden may persist or evolve despite catheter ablation.
- Implantable cardioverter-defibrillator decisions should be based on integrated risk stratification.

## Introduction

Arrhythmogenic cardiomyopathy (ACM) is an inherited myocardial disorder characterized by progressive fibro-fatty replacement and an increased risk of ventricular arrhythmias and sudden cardiac death.^1^ Contemporary evidence expands its traditional right ventricular phenotype to include biventricular forms, frequently driven by desmosomal variants such as PKP2.^2,3^ Although arrhythmias are classic hallmarks, atypical presentations with advanced structural disease can mimic dilated cardiomyopathy (DCM) or myocarditis.^4^ We report a genetically confirmed *PKP2*-associated biventricular ACM case where severe heart failure and systemic thromboembolism dominated the clinically apparent presentation, emphasizing key diagnostic and therapeutic challenges.

## Summary figure

**Figure:**
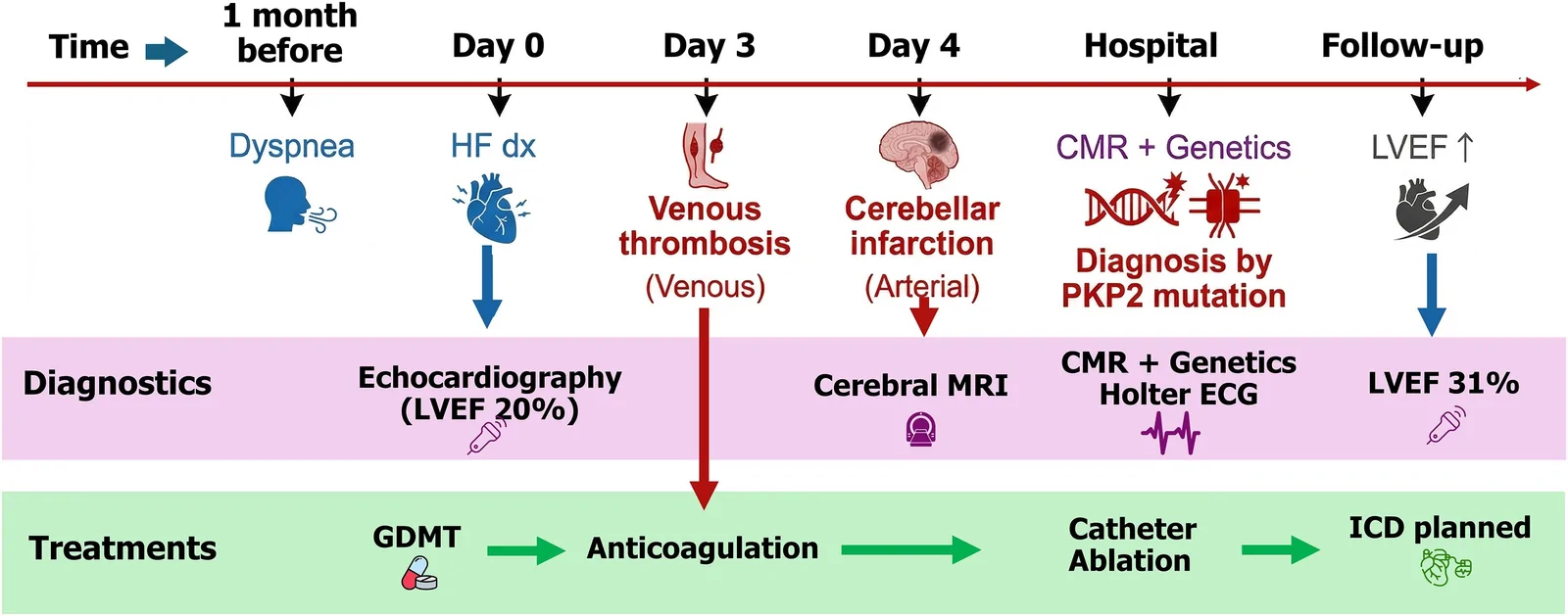
Timeline of clinical events (Biventricular Arrhythmogenic Cardiomyopathy with *PKP2* Mutation). Schematic representation of the clinical course from initial symptoms to follow-up. The patient presented with progressive heart failure, followed by venous thrombosis and subsequent cerebellar infarction despite anticoagulation. Multimodality imaging and genetic testing confirmed the diagnosis of arrhythmogenic cardiomyopathy due to a *PKP2* mutation. Treatment included guideline-directed medical therapy, anticoagulation, and catheter ablation, resulting in partial reverse remodelling.

## Case presentation

A 22-year-old previously healthy woman presented with progressive exertional dyspnoea over 1 month. She had no history of oral contraceptive use, pregnancy, trauma, prolonged immobilization, recent infections (including COVID-19), or prior central venous catheterization. Physical examination revealed bilateral lower limb oedema and orthopnoea. Initial electrocardiography (ECG) demonstrated sinus rhythm without arrhythmias, inverted T waves in leads V1–V3 and low QRS voltages in limb leads. Echocardiography showed right ventricular dilation with regional aneurysmal changes and severe left ventricular systolic dysfunction (LVEF 20%) without macroscopic intracardiac thrombus.

On hospital day three, she developed right upper extremity deep vein thrombosis. Extensive workups, including antiphospholipid antibodies and multimodality imaging (chest computed tomography [CT] and ultrasounds), strictly ruled out primary thrombophilia, autoimmune diseases, occult malignancies, and thoracic outlet syndrome. Despite initiating therapeutic enoxaparin, she soon suffered an acute left cerebellar infarction. She was subsequently managed with guideline-directed medical therapy (GDMT) and long-term anticoagulation with rivaroxaban.

**Figure 1:**
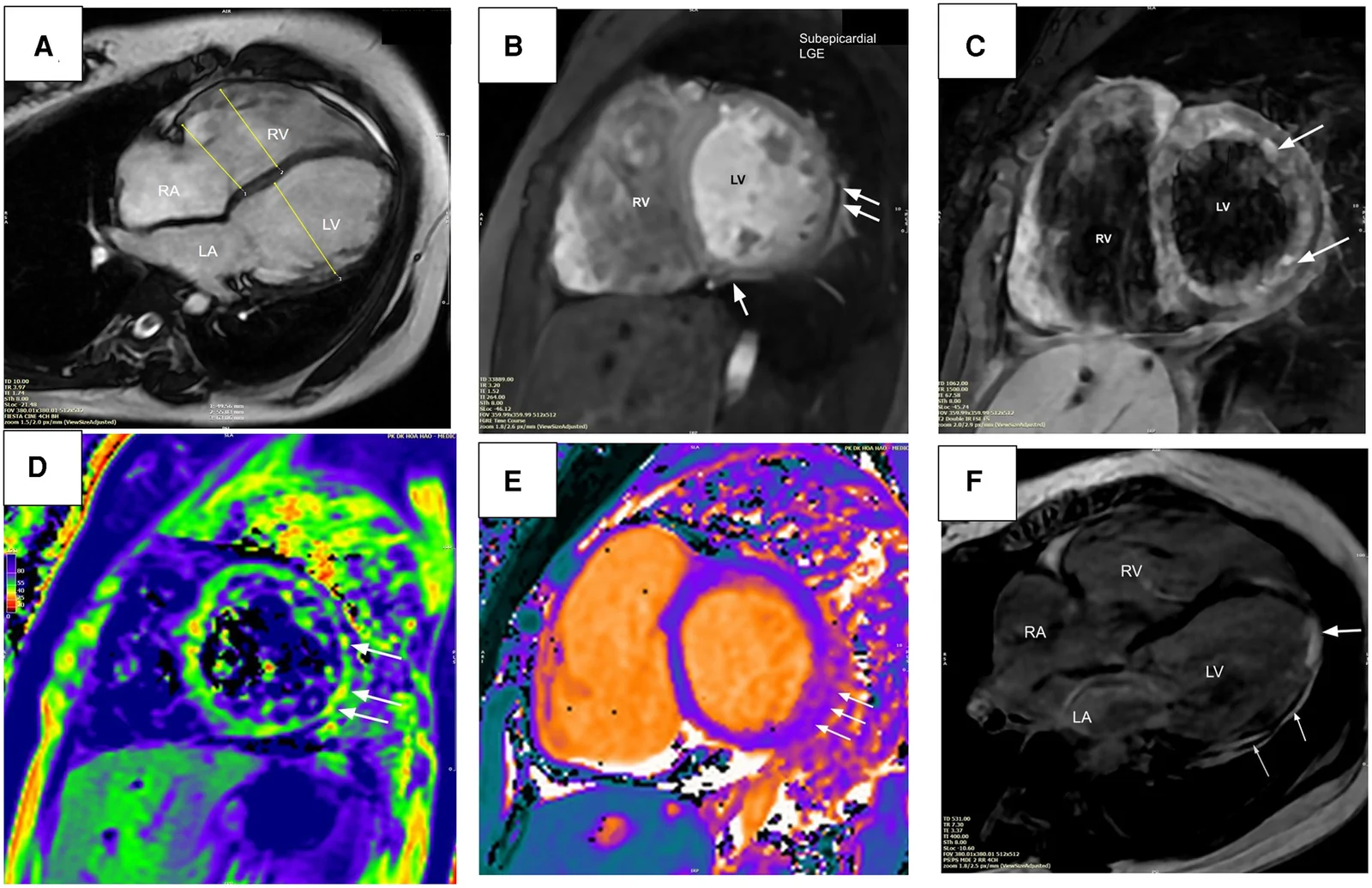
Cardiac magnetic resonance imaging. (*A*) Four-chamber cine view demonstrating biventricular dilation with severely reduced systolic function. (*B*) Late gadolinium enhancement short-axis view showing patchy subepicardial and mid-wall enhancement (arrows), predominantly involving the lateral wall and right ventricular insertion point. (*C*) T2-weighted double inversion recovery imaging demonstrating increased myocardial signal intensity (arrows), consistent with myocardial oedema. (*D*) T2 mapping demonstrating increased myocardial T2 values (arrows), predominantly involving the lateral and inferolateral walls. Elevated native T2 relaxation times (Native T2 = 68 ms) (*E*) Native T1 mapping showing elevated myocardial T1 values (arrows), consistent with diffuse fibrosis. Quantitative analysis demonstrated elevated native T1 (1107 ms) and extracellular volume (42%). (*F*) Long-axis late gadolinium enhancement view confirming subepicardial enhancement involving the lateral wall, supporting a non-ischaemic pattern of myocardial fibrosis.

Cardiac magnetic resonance (CMR) imaging demonstrated marked biventricular involvement. Using Simpson's rule for volumetric analysis, the right ventricle was dilated with severely reduced systolic function (right ventricular systolic dysfunction 10%), alongside a moderately dilated left ventricle with global hypokinesia (LVEF 20%) (see Supplementary material online, *Video S1*). Late gadolinium enhancement (LGE) revealed a strictly non-ischaemic pattern of patchy subepicardial and mid-wall fibrosis (total scar burden 8 g; approximately 9% left ventricle mass) involving the anterior and lateral walls, including the right ventricular insertion point (see Supplemantary material online, *Video S2*). Myocardial perfusion imaging showed no perfusion defects in the left ventricular walls (see Supplementary material online, *Video S3*). Elevated native T1 and T2 values further supported diffuse fibrosis and concurrent myocardial injury (*Figure 1*).

**Figure 2:**
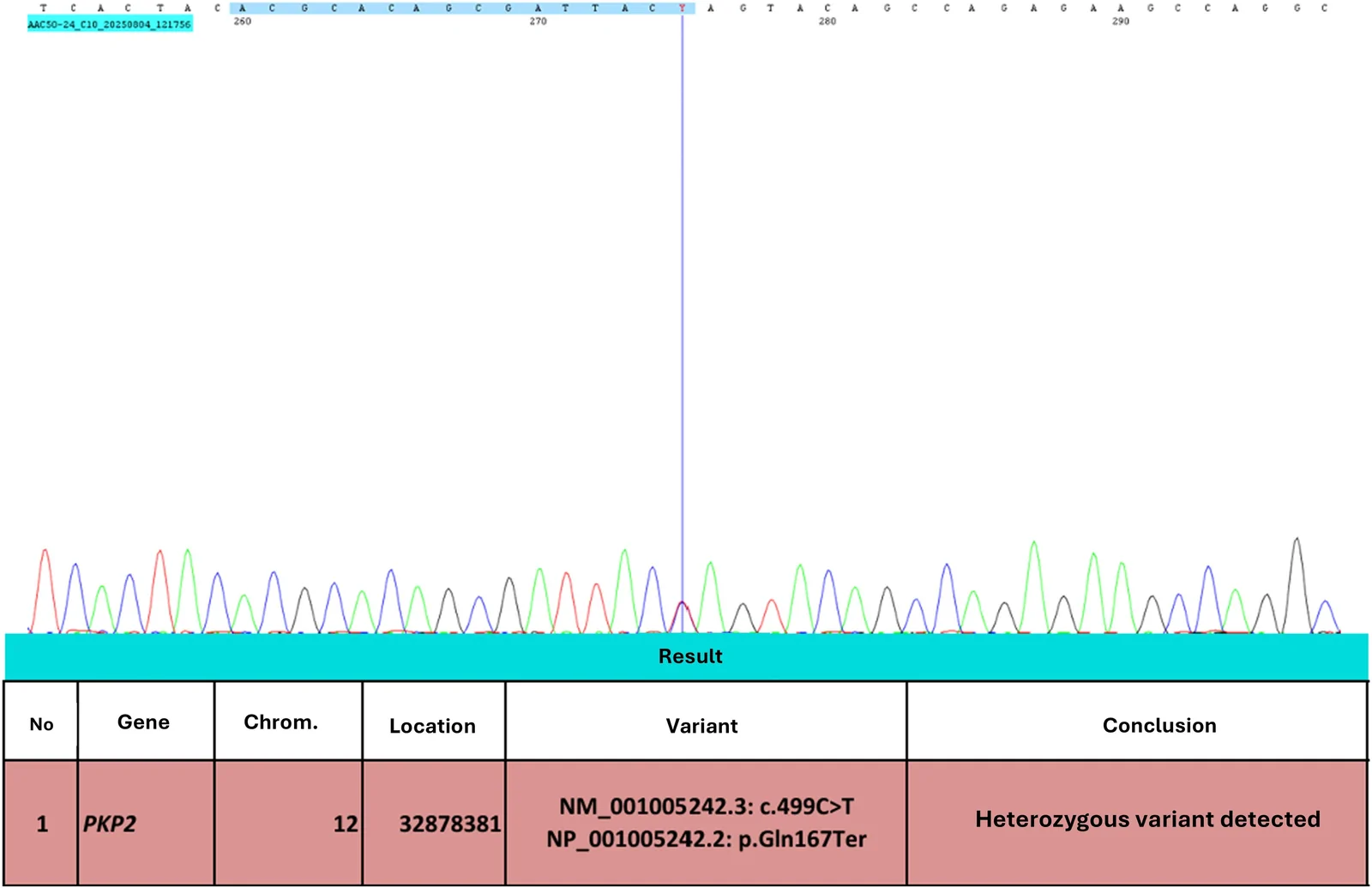
Heterozygous gene *PKP2* mutation. The p.Gln167Ter variant is classified as pathogenic based on established the American College of Medical Genetics and Genomics and the Association for Molecular Pathology (ACMG/AMP) criteria and supports the diagnosis of arrhythmogenic cardiomyopathy.

**Figure 3:**
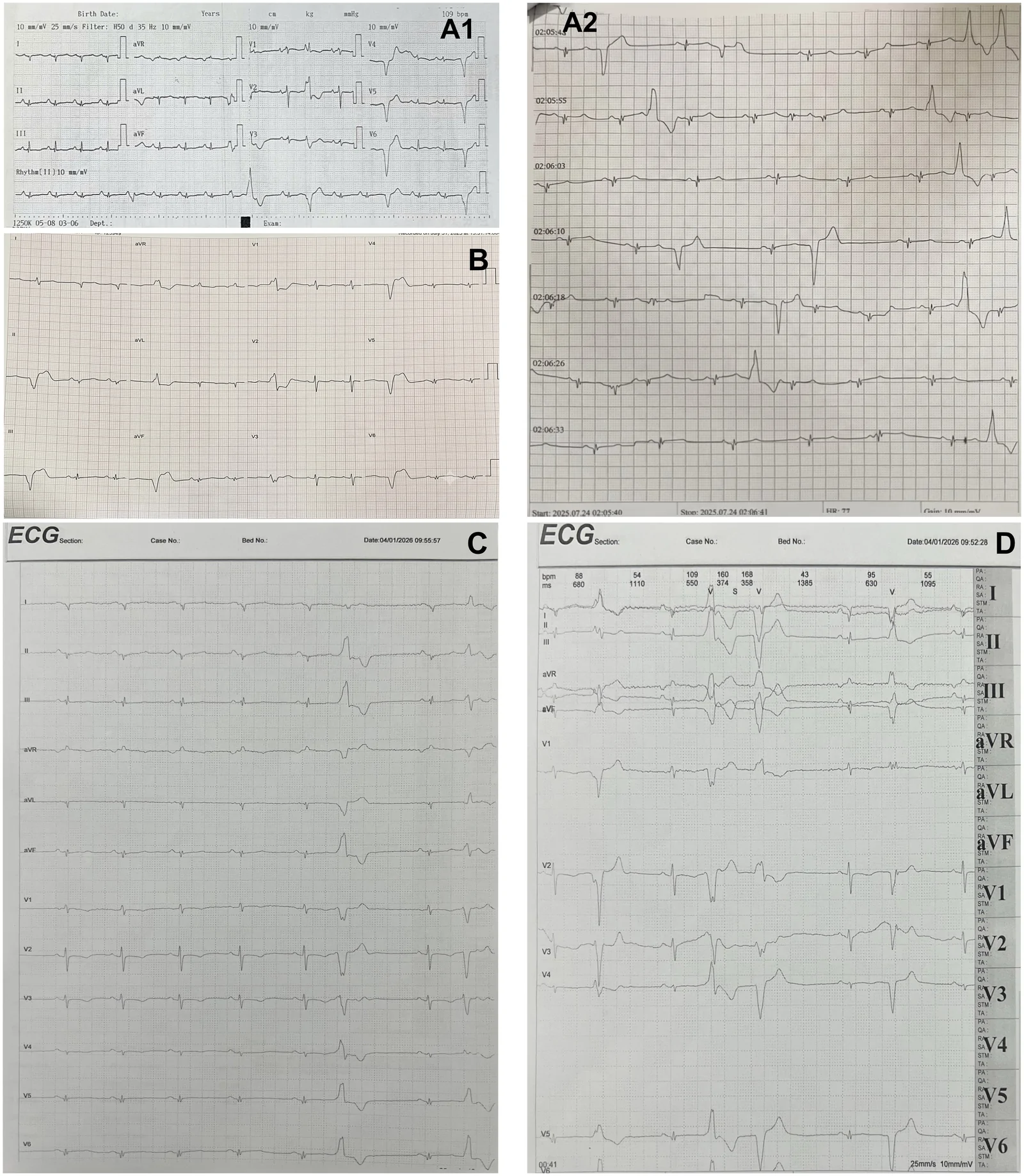
Electrocardiographic timeline. (*A*) A 12-lead electrocardiography and Holter electrocardiography before ablation demonstrating a high burden of premature ventricular complexes (15.7%), inverted T waves in leads V1–V3 (a major repolarization criterion), low QRS voltages in limb leads (a minor criterion for left ventricular involvement). (*B*) Post-ablation electrocardiography demonstrating persistent premature ventricular complexes at rest and an increased burden during ambulation, including ventricular couplets. (*C* and *D*) Follow-up electrocardiography demonstrating persistent premature ventricular complexes at rest and an increased burden during ambulation, including ventricular couplets.

Genetic testing identified a heterozygous pathogenic nonsense variant in the *PKP2* gene (NM_001005242.3: c.499C > T; p.Gln167Ter), consistent with a loss-of-function mechanism (*Figure 2*). Cascade screening confirmed autosomal dominant inheritance; the identical variant was found in the patient's mother, but not her younger sister. Despite her positive genotype, the mother's comprehensive cardiovascular screening (ECG, Holter, and echocardiography) was completely unremarkable. This genotype-positive/phenotype-negative status perfectly illustrates the incomplete penetrance characteristic of *PKP2*-associated ACM, prompting her enrolment in long-term surveillance while her sister was safely discharged. In June 2025, Holter monitoring revealed a high multifocal premature ventricular contraction (PVC) burden (15.7%) and NSVT (*Figure 3*). Electrophysiological mapping identified arrhythmic foci originating from the right ventricular outflow tract (RVOT) and the aortomitral continuity. RVOT ablation was successful but left-sided ablation was deferred due to the high risk of myocardial perforation in the structurally compromised myocardium.

Following the clinical exclusion of coronary artery disease and systemic autoimmune conditions, invasive endomyocardial biopsy and ^18^Flurodeoxygluco-Positron Emission Tomography [^18^FDGPET imaging were safely deferred. A ‘definite’ diagnosis of biventricular ACM was established using the 2020 Padua Criteria, rigorously integrating her severe biventricular morpho-functional changes, non-ischaemic CMR pattern, arrhythmic burden, and the pathogenic *PKP2* variant.^2^

The patient tolerated all interventions well and remained adherent to medical therapy without adverse events. Follow-up echocardiography demonstrated partial reverse remodelling, with improvement of LVEF to 31%. However, electrocardiography continued to show persistent PVCs at rest and an increased arrhythmic burden with couplets during ambulation, indicating ongoing electrical instability. Given the combination of severe structural disease, evolving arrhythmias, and a pathogenic *PKP2* variant, implantable cardioverter-defibrillator implantation was planned for primary prevention of sudden cardiac death after multidisciplinary evaluation.

## Discussion

This case illustrates an expanded and atypical clinical presentation of *PKP2*-associated ACM. While traditionally considered a right ventricular disease, our patient's clinically apparent presentation was dominated by severe biventricular failure and thromboembolism. Classic arrhythmic hallmarks, including NSVT and a high PVC burden, did not prompt initial medical attention and were only documented on later surveillance.^2,3^

There is a profound phenotypic overlap between biventricular ACM, genetic DCM, cardiac sarcoidosis, and inflammatory cardiomyopathies, which frequently leads to diagnostic delays. Our patient initially presented in the ‘dilated phase’ of ACM, where progressive fibrofatty replacement extends extensively into the left ventricle, causing profound systolic dysfunction (LVEF 20%) that perfectly phenocopies DCM. Furthermore, the subepicardial and ring-like LGE pattern observed on CMR, along with myocardial oedema (elevated T1/T2 values), while highly suggestive of biventricular ACM, are also classic hallmarks of active myocarditis or cardiac sarcoidosis, creating a well-described diagnostic trap. It is increasingly recognized that patients with desmosomal mutations may experience ‘hot phases’ characterized by recurrent episodic myocardial inflammation and oedema, further blurring the lines with acute inflammatory cardiomyopathy. In this context, integrating genetic testing was paramount. Identifying the *PKP2* nonsense mutation not only corrected the misdiagnosis, obviating the need for invasive endomyocardial biopsy or ^18^F-FDG PET imaging, but also enabled cascade screening.

*PKP2* encodes plakophilin-2, a critical component of the macromolecular ‘connexome’ complex at the intercalated disc.^5^ Loss-of-function variants promote electrical remodelling and progressive fibrofatty replacement. Although electrical abnormalities classically precede structural disease, the initial presentation in our patient was dominated by advanced heart failure and thromboembolism. Her overt high arrhythmic burden was likely unmasked later only when exacerbated by profound structural damage and regional aneurysms.

A notable feature of this case is the simultaneous venous and arterial thromboembolism in a young patient lacking typical risk factors or atrial fibrillation. While paradoxical embolism via a patent foramen oval was not definitively ruled out, the most plausible mechanism is the profound global stasis resulting from severe biventricular failure. In ACM, the extensive structural remodelling and depressed contractility (LVEF 20%) create a highly prothrombotic environment, independently driving both right-sided venous thrombosis and left-sided cardio embolism events without initial macroscopic thrombi.^6^ Additionally, myocardial inflammation may contribute to endothelial dysfunction and a prothrombotic state. However, current evidence supporting routine anticoagulation in ACM patients without atrial fibrillation or documented intracardiac thrombus remains limited, highlighting an area requiring further investigation.^1,7^ Although severe biventricular dysfunction may plausibly contribute to thrombosis through blood stasis and adverse chamber remodelling, the thromboembolic events observed in this patient should be interpreted as an association rather than a definitive manifestation of ACM itself.

This case also emphasizes that arrhythmic manifestations in ACM may evolve over time and may not represent the initial clinical presentation. Although catheter ablation effectively reduced ventricular ectopy, it did not eliminate the underlying arrhythmogenic substrate, as evidenced by persistent and exertion-induced arrhythmias on follow-up.

From a therapeutic perspective, GDMT contributed to partial reverse remodelling,^8^ while catheter ablation reduced arrhythmic burden. Given the patient's persistent ventricular ectopy, severe ventricular dysfunction, and pathogenic *PKP2* variant, an implantable cardioverter-defibrillator (ICD) was planned for primary prevention. This multiparametric decision aligns with contemporary guidelines for sudden cardiac death risk stratification.^7^ Following multidisciplinary care, the patient reported symptomatic improvement, accepted the long-term management strategy, and was advised to strictly avoid competitive and high-intensity endurance sports.

## Conclusion

Arrhythmogenic cardiomyopathy may present with advanced biventricular dysfunction and thromboembolism as the primary clinically apparent features, while significant arrhythmias are documented on later surveillance. Recognizing these atypical presentations requires integrating multimodality imaging, genetic testing, and serial electrocardiography to definitively establish the diagnosis, guide management, and direct sudden cardiac death prevention strategies, including ICD therapy.

## Lead author biography


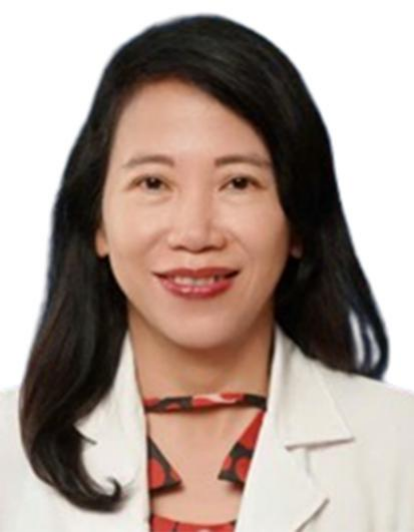
Dr Binh Thi Thanh Dao is a cardiologist in Ho Chi Minh City, Viet Nam. She obtained her PhD in 2007 and has extensive experience in clinical cardiology. Her primary areas of interest include cardiomyopathies, cardiac imaging, and arrhythmia management. Her academic work focuses on integrating imaging and genetic data to enhance diagnostic accuracy and risk stratification in cardiovascular diseases. She has contributed to several clinical studies and case reports aimed at improving the understanding of complex cardiac conditions.

## Supplementary Material

ytag636_Supplementary_Data

## Data Availability

The data underlying this article are available in the article. Further de-identified data are available from the corresponding author upon reasonable request.
